# Sex Differences in Mitochondrial Function Following a Controlled Cortical Impact Traumatic Brain Injury in Rodents

**DOI:** 10.3389/fnmol.2021.753946

**Published:** 2021-10-13

**Authors:** Olivia J. Kalimon, Patrick G. Sullivan

**Affiliations:** ^1^Department of Neuroscience, University of Kentucky, Lexington, KY, United States; ^2^Spinal Cord and Brain Injury Research Center, University of Kentucky, Lexington, KY, United States; ^3^Lexington VA Healthcare System, Lexington, KY, United States

**Keywords:** bioenergetics, oxidative stress, glucose utilization, sex hormone influence, CNS injury

## Abstract

Traumatic brain injury (TBI) is a complex disease to study due to the multifactorial injury cascades occurring after the initial blow to the head. One of the most vital players in this secondary injury cascade, and therapeutic target of interest, is the mitochondrion. Mitochondria are important for the generation of cellular energy, regulation of cell death, and modulation of intracellular calcium which leaves these “powerhouses” especially susceptible to damage and dysfunction following traumatic brain injury. Most of the existing studies involving mitochondrial dysfunction after TBI have been performed in male rodent models, leaving a gap in knowledge on these same outcomes in females. This mini-review intends to highlight the available data on mitochondrial dysfunction in male and female rodents after controlled cortical impact (CCI) as a common model of TBI.

## Introduction

### Clinical Overview of Sex Differences in Traumatic Brain Injury

Traumatic brain injury (TBI) occurs from a primary bump, blow, jolt, or penetrating injury to the head that disrupts brain function. TBI is a continuously growing health concern as the most recent CDC data from 2019 showed 61,000 TBI-related deaths in the US (Centers for Disease Control and Prevention, 2020). Following the primary impact on the head, there are complex biochemical, cellular, and molecular secondary injury cascades that cause, ischemia, excitotoxicity, cerebral swelling, inflammation, and diffuse axonal injury (Kochanek et al., 2000). In humans, TBI can be classified as mild, moderate, or severe depending on the clinical presentation of neurological symptoms including Glasgow Coma Scale (GCS) score, headache, dizziness, loss of consciousness, seizures, coma, or even death and these symptoms vary from person to person.

Gupte et al. (2019) nicely summarized the available data on the reported outcomes in males vs. females in human and animal TBI studies. One of the main takeaway points from that review was that a larger percentage of the total human studies reported women faring worse than men after TBI, conversely, a larger percentage of the total rodent studies reported females having better outcomes than males. However, when the human and rodent data were separated by injury severity (mild-moderate-severe), a larger percentage of studies reported both women and females had better outcomes than men/males after a more severe injury, but results after mild TBI are less conclusive. Data assessing outcomes after mild TBI showed a higher percentage of studies reported women experienced worsened outcomes and females had mixed results compared to their male counterparts (Gupte et al., 2019). The authors summed up that sex differences in TBI can be considered a “mosaic” where women/females have better outcomes in certain measures and men/males have better outcomes in others, driving home the importance of studying both sexes. The 2016 CDC TBI Surveillance Report identified males having significantly higher age-adjusted rates of TBI-related hospitalization compared to females, but the highest number of hospitalizations occurred at age >75 years old (Center for Disease Control and Prevention, 2021). This sex difference may be attributed to men being more involved in contact sports, military service, or even physical altercations (Biegon, 2021). Further, when sex and age are considered after moderate-severe TBI, results have shown that men between 15–50 years of age had a slightly increased survival rate over age-matched women. However, this effect is reversed after ~50 years of age, showing peri- and post-menopausal women (the years around or after menopause when ovarian hormone levels are diminishing) had increased survival over age-matched men (Cleveland Clinic, 2019; Biegon, 2021). A conflicting study was performed in 13,437 patients with moderate-severe TBI in which the authors found men had similar mortality rates as women after TBI. However, when the data is separated by age, pre-menopausal women (<50 years old) had similar mortality rates as age-matched men, but post-menopausal women (≥50 years old) had reduced mortality compared to age-matched males. This study concluded that ovarian hormones present during the pre-menopausal period (e.g., estradiol and progesterone) are not neuroprotective against TBI-related mortality (Davis et al., 2006). The risk of TBI in the female population is growing given the increasing number of women serving in active military duty, and participating in competitive sports like soccer and basketball, as well as women suffering from intimate partner violence (IPV) who often receive severe blows to the head (Arambula et al., 2019; Gupte et al., 2019). Unfortunately, there are still knowledge gaps on the effects of hormonal status at the time of injury, safety, and efficacy of TBI interventions for women, and the importance of risk factors associated with poor outcomes after TBI in women (Biegon, 2021). As detrimental as this disease is, there are currently no FDA-approved pro-regenerative or neuroprotective therapeutics for men or women after TBI, though there are currently eight active, not recruiting and 22 recruiting interventional Phase I, II, and III clinical trials throughout the US (NIH U.S. National Library of Medicine, 2021a,b).

One trial of note is the 2014 Phase III clinical trial involving progesterone as a treatment for acute TBI. This double-blind, placebo-controlled trial was stopped for futility because intravenous progesterone was not demonstrating improved neuropsychological performance or higher Glasgow Coma Scale Extended (GOSE) scores in patients with moderate-severe TBI (Goldstein et al., 2017). Previously, progesterone treatment showed promise in animal studies of TBI which led to an early phase II trial with 100 patients enrolled with 77 subjects randomized to receive intravenous progesterone and 23 to receive placebo. Results from this early human study showed reduced 30-day mortality in the progesterone group compared to placebo and similar rates of adverse events between progesterone and placebo groups, suggesting that progesterone administration is safe and provides neuroprotective effects (Wright et al., 2007). Progesterone was first noted as a topic of interest in 1987 by Attella et al. (1987) who saw reduced edema in pseudopregnant rats compared to normal-cycling rats after TBI. It was hypothesized these observed differences were due to increased levels of gonadal hormones during pseudopregnancy compared to control rats (Attella et al., 1987). Failure of progesterone in the larger clinical trial may have been due to increasing the sample size. The review by Gupte et al. (2019) found that 53% of studies with 0–1,000 patients reported women having worse outcomes than men, but in studies with >10,000 patients, 67% reported better outcomes in females than males (Wright et al., 2007). Ultimately, the failure of progesterone, like many other failed TBI trials, is most likely due to the complexity and heterogeneity of TBI, the unidimensional characterization of injury in humans, and the limited ability to translate experimental data to human TBI (Saatman et al., 2008; Skolnick et al., 2014). For these reasons, it is imperative to utilize different injury models to understand the underlying pathophysiology of TBI and ultimately translate those findings into humans. However, this mini-review focuses on the controlled cortical impact (CCI) model of TBI and the effect of this injury on various mitochondrial outcomes in male and female rodents.

### Estrogen Receptors and Mitochondria

Sex differences in mitochondrial function cannot be addressed without first discussing sex hormones and their receptors. Unfortunately, little is known about the role of mitochondrial progesterone receptors in the brain, though it is documented to influence mitochondrial respiration in the heart (Dai et al., 2013). It is typically believed both estrogen and progesterone receptors act through genomic mechanisms to regulate mitochondria, though the addition of estradiol directly to isolated hepatic mitochondria was shown to reduce peroxide production and limit mitochondrial cytochrome c release (Borrás et al., 2010). This study suggests estradiol acts directly on mitochondrial estrogen receptors (ERs) to protect against oxidative stress. Within neurons, ERs have been found in the nucleus, plasma membrane, and mitochondria, though it is unknown whether ER action is coordinated between these compartments or if ER signaling remains independent within these compartments (Brinton, 2008). Evola et al. (2018) measured ERβ content in total hippocampal and cortical mitochondria between normal, aging male and female rats. They found no significant difference in ERβ content between sexes in any of the age groups, though there was a trend of females having greater ERβ content in the cortex at 4 weeks old compared to males (Evola et al., 2018). In a rat model of diffuse TBI, vehicle-treated OVX female rats showed decreased ERβ mRNA expression in the whole brain after injury than estradiol-treated rats (Khaksari et al., 2015). Interestingly, protein levels of ERβ were not significantly different between the groups. Both mRNA and protein levels of ERα were significantly increased with estradiol treatment after TBI, though TBI alone or vehicle treatment after TBI were not different from sham ERα mRNA or protein expressions (Khaksari et al., 2015). These studies were only conducted in female rats after experimental TBI though, so studies should be conducted in male rats as well to determine if there is sexual dimorphism. Additionally, these studies should be observed in mitochondrial fractions rather than the whole brain to determine whether expression changes after TBI affect mitochondrial function after injury.

### Estrogen and Progesterone Influence on Mitochondrial Bioenergetics

**Figure 1:**
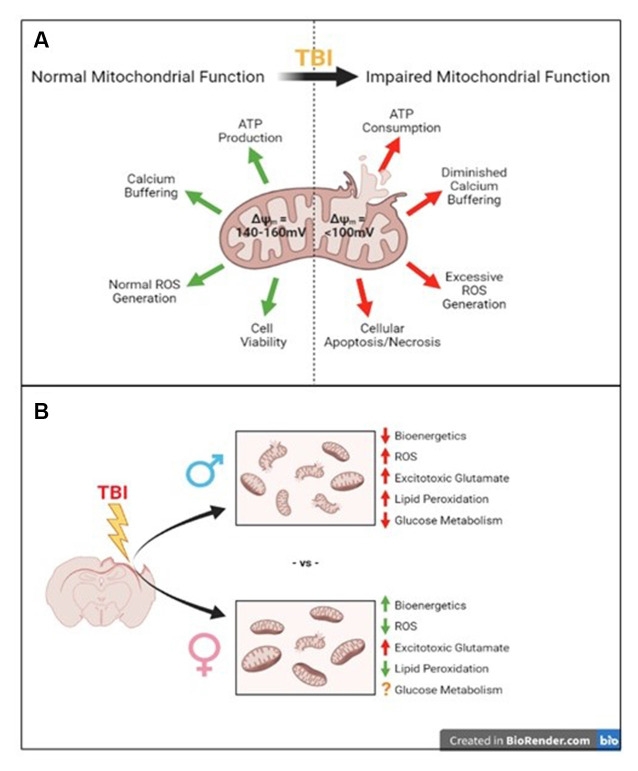
Summary of traumatic brain injury-induced mitochondrial disruptions compared to their normal functions and what we know so far about sex-specific differences in these disruptions. **(A)** Normal mitochondrial function is disrupted after traumatic brain injury (TBI). **(B)** The well-known consequences of TBI such as impaired mitochondrial bioenergetics, increased reactive oxygen species (ROS), increased excitotoxicity and lipid peroxidation, and impaired glucose metabolism have been established in male models of injury. The few studies addressing these in female models have shown increased bioenergetics, reduced ROS, increased acute glutamate levels, and reduced lipid peroxidation, though glucose metabolism is still unclear compared to males.

Notably, mitochondria are important targets of secondary injury caused by TBI. Known as the “powerhouse” of the cell, mitochondria are the primary location of ATP synthesis, but their maintenance of cellular homeostasis drives home their nickname. Mitochondria are responsible for regulating cell death pathways, calcium buffering, oxidative phosphorylation, and ATP synthesis as summarized in Figure 1A, which makes them important targets in neurological disorders like Parkinson’s disease, Alzheimer’s disease, TBI, and stroke (Yonutas et al., 2016). A study by Gaignard et al. (2015) found gonadal status influences mitochondrial function in female mice, but not males. Ovariectomy (OVX) impaired coupled and maximal NADH-linked respiration after addition of pyruvate + ADP and the uncoupler m-Cl-CCP in isolated brain mitochondria compared to intact females; however, castration in male mice did not impair oxygen consumption rates compared to intact males (Gaignard et al., 2015). Additionally, intact females had higher coupled and maximum NADH-linked respiration compared to intact males, but OVX knocked these rates down to male levels (Gaignard et al., 2015). This study indicates female sex hormones, not male hormones, affect brain mitochondrial respiratory function, making it an important topic to study in the scope of TBI. To determine the role of estrogen (30 μg/kg) and progesterone (30 μg/kg) on isolated whole brain mitochondria from uninjured, OVX rats, Irwin et al. (2008) measured bioenergetic function by respiratory control ratio (RCR) after a single *in vivo* exposure. RCR is defined by dividing State III (coupled respiration after addition of ADP) by State IV (proton leak after addition of ATP synthase inhibitor, oligomycin) respiration to determine mitochondrial coupling of the electron transport system to oxidative phosphorylation and general mitochondrial health. They found *in vivo* progesterone treatment alone gave a 24.5% increase in mitochondrial RCR and estradiol gave a 13.4% increase in RCR, both compared to mitochondria from vehicle-treated rats (Irwin et al., 2008). This study was performed in naïve, uninjured rats, but shows the presence or absence of female sex hormones affects mitochondrial bioenergetic function.

The studies mentioned previously in this section were performed in mitochondria isolated from uninjured animals; now we’ll shift focus to studies analyzing bioenergetics in brain mitochondria isolated from rodents that have received controlled cortical impact (CCI) injury. CCI is a model of TBI which applies a mechanical force directly to the exposed cortex by a pneumatic piston or electromagnetic actuator creating morphologic, cerebrovascular, and neurobehavioral impairments similar to those observed in humans (Osier and Dixon, 2016; Yonutas et al., 2016). Greco et al. (2020) aimed to determine how cerebral metabolic state influences substrate metabolism after severe CCI in male and female rats. The authors measured State III, State IV, and State V (maximum respiration after addition of the mitochondrial uncoupler, FCCP) respiration, as well as the RCR in mitochondria isolated from the ipsilateral peri-contusional cortex. They administered different metabolic substrates [glucose, lactate, β-hydroxybutyrate (BHB)] in the hyper- (0–3 h post-injury; early administration) or hypo-glycolytic (6–9 h post-injury; late administration) state and measured the effects on bioenergetic activity. Male rats showed reduced State III respiration and reduced RCR 24-h post-severe CCI compared to sham. The female rats showed reduced State III and State V respiration, however, RCR was not significantly different from sham, meaning general mitochondrial bioenergetic health was not impaired by the injury. Overall, it was shown that late lactose provided benefit in both males and females while interestingly, early BHB was beneficial to male mitochondria after injury, but early and late BHB administration impaired female mitochondrial function (Greco et al., 2020). This may be due to estrogen’s role in promoting glucose utilization and mitochondrial bioenergetic function as reported in healthy neurons, preventing the shift to alternative fuel sources and hypometabolism seen in Alzheimer’s Disease, though this has yet to be fully addressed after TBI (Brinton, 2008). In all, one important finding from this study was that the normal cycling female rats did not have impaired RCR after severe CCI, which may indicate cycling ovarian hormones play a protective role in maintaining mitochondrial health after injury, though this has yet to be replicated or examined further.

A study by Robertson et al. (2006) looked at the effects of progesterone treatment on mitochondrial function in female ovariectomized (OVX) rats after moderate-severe CCI. They found that 1 h after injury, mitochondria from the progesterone group (25 ng/ml) had restored RCR to sham levels compared to injured-vehicle control (Robertson et al., 2006). Interestingly, this study saw reduced RCR after injury compared to sham, an effect not seen by Greco et al. (2020) suggesting this injury effect is present after elimination of ovarian hormones by OVX. The effect of progesterone was further studied in male and female rat models of pediatric TBI by the same group (Robertson and Saraswati, 2015). They found progesterone treatment after CCI improved mitochondrial RCR compared to vehicle-treated male pups. This reduction came from decreased State IV respiration as there was no change in State III respiration between the groups. In the female pups, however, there were no significant alterations in State III, State IV, and RCR in the progesterone- or vehicle-treated female pups after CCI than the normal, uninjured pups (Robertson and Saraswati, 2015). This study suggested progesterone may be applied for the treatment of pediatric TBI, though progesterone treatment failed to provide benefits in adults after TBI (Robertson and Saraswati, 2015; Goldstein et al., 2017).

### Testosterone Effect on Mitochondrial Bioenergetics After CCI

Although most mitochondrial research utilizes male models of TBI, there is little known about the influence of testosterone (TS) on mitochondrial bioenergetics after injury. TS deprivation in castrated male rats was shown to impair mitochondrial gene expression in the hippocampus, which suggests genomic action of TS plays a role in maintaining normal mitochondrial function (Hioki et al., 2014). A study by Carteri et al. (2019) hypothesized that mitochondrial dysfunction is influenced by the reduction of brain TS levels after injury and that supplementation of TS would improve mitochondrial function. The authors observed improved mitochondrial bioenergetics in the whole cortex of male mice treated with testosterone (TS) compared to the vehicle after severe CCI (Carteri et al., 2019). TS was administered 24 h after CCI and *ex vivo* cortical mitochondrial analyses occurred at 10-days post-CCI. They found mitochondrial membrane potential was significantly elevated at baseline compared to sham but was restored to sham levels by TS administration. Membrane potential is a large driver of mitochondrial action, so the authors then looked at the effect of TS administration on oxidative phosphorylation (OxPHOS) after CCI. State III respiration (pyruvate, malate, glutamate, and ADP) was significantly decreased in the injured-vehicle mice compared to injured-TS and vehicle-sham mice. Maximum OxPHOS through complex-I and II activation after addition of State III substrates, succinate, and saturating ADP was also reduced in injured-vehicle mice compared to vehicle-sham and injured-TS mice. The authors concluded TS treatment after CCI maintained mitochondrial bioenergetic function to promote cell survival, though this has yet to be explored by other groups (Carteri et al., 2019).

### Excitotoxicity, Oxidative Stress, and Lipid Peroxidation

The brain requires a lot of energy to maintain normal neuronal function, so this high metabolic demand needs to be fulfilled by mitochondria and oxygen supply, making neurons sensitive to mitochondrial oxidative damage (Irwin et al., 2008). Following TBI, several secondary injury mechanisms occur including excitotoxicity and oxidative stress. In short, after the initial injury, there is increased excitotoxic glutamate that binds to AMPA and NMDA receptors causing an influx of calcium to the neuron. Mitochondria are responsible for sequestering calcium, however, eventually, the load becomes too much and further contributes to the excitotoxic event. Mitochondrial membrane potential decreases to dangerous levels (100–120 mV) ramping up the ETC to restore membrane potential to healthy levels (180 mV), but in doing so, increases the propensity for electron slippage and reactive oxygen and nitrogen species (ROS/RNS) production. If the membrane potential gets too low, the ATP synthase will start consuming ATP. Eventually, the membrane potential is so low that the mitochondrial permeability transition pore (mPTP) opens and releases proteins like cytochrome c to signal for apoptosis (Yonutas et al., 2016). Radical production damages lipid membranes by reacting with polyunsaturated fatty acids in a process known as lipid peroxidation, a well-established contributor to TBI neuropathology (Hill et al., 2020).

Borrás et al. (2003) have shown synaptic and non-synaptic brain mitochondria isolated from intact female rats generate less hydrogen peroxide than intact males and ovariectomized (OVX) females. Hepatic mitochondria from OVX rats administered daily with 17β-estradiol (1 μg/kg body weight) had peroxide levels that were not statistically different from intact female mitochondria; it was shown this antioxidant effect of estradiol correlated with higher glutathione levels (Borrás et al., 2003). These studies were not performed in brain mitochondria, though it shows proof of principle that estrogen has antioxidant effects on hepatic mitochondria (Borrás et al., 2003). It has been shown in male models of TBI that injury increases mitochondrial reactive oxygen species (ROS) production (Kumar Sahel et al., 2019; Greco et al., 2020). A study by Greco et al. (2020) also showed increased peroxide production 24 h post-CCI in male rats consistent with previous data; though notably there was no difference in injured female peroxide production compared to sham. Robertson and Saraswati (2015) reported progesterone (10 mg/kg) administration 1 h post-CCI did not prevent the injury-induced decrease in glutathione in brain mitochondria isolated from female rats but did prevent this decrease in males. Notably, mitochondria from uninjured females had significantly higher glutathione levels than uninjured males. This study, however, was performed in a pediatric model of CCI though it indicated progesterone administration after an injury is not neuroprotective against oxidative stress in adolescent females, but may benefit males (Robertson and Saraswati, 2015). A study by Carteri et al. (2019) showed testosterone (TS) administration reduced baseline cortical peroxide production after CCI relative to sham levels compared to the vehicle-treated injured mice. However, the rate of peroxide production from TS-treated mice was elevated relative to sham after various respiratory substrate administrations, indicating TS does not completely prevent mitochondrial peroxide production after CCI. Additionally, the injured-vehicle mice had elevated total ROS levels compared to injured-TS and vehicle-sham mice (Carteri et al., 2019). These studies indicate endogenous female hormones present during the impact and testosterone administration after the impact may provide neuroprotection from oxidative stress in rodents.

A clinical study assessing excitotoxic glutamate levels in men and women after severe TBI found men had elevated cerebral spinal fluid (CSF) glutamate levels during every 12 h observation period after injury (0–12, 12–24, 24–36, and 36–48 h after injury; Wagner et al., 2005). CSF glutamate remained low in the female group during the first interval, peaked in the second interval above males, and continuously decreased until 48 h. The authors suggested this was due to estrogen attenuation of NMDA-mediated calcium activity in response to the excitotoxic glutamate levels seen after severe TBI, though they agreed this relationship needed to be further addressed (Wagner et al., 2005). Another study observed the lipid peroxidation marker F_2_-isoprostane in human CSF after severe TBI found women had markedly lower F_2_-isoprostane levels compared to men (Bayir et al., 2004). These studies highlight the observed sex differences in oxidative stress seen in the clinic and reveal antioxidant effects of female sex hormones (i.e., estrogen and progesterone) in these outcomes.

### Glucose Utilization After TBI

The brain has high metabolic demand and preferentially utilizes glucose for energy, so glucose availability is vital for normal brain function. Cerebral autoregulation maintains normal cerebral blood flow(CBF) across a range of blood pressures to ensure sufficient oxygen and glucose can get to their destinations. Cerebral autoregulation is impaired after TBI causing CBF to decrease, which often leads to poor patient outcomes making this one of the important targets for therapeutic intervention following TBI (Roof and Hall, 2000; Jalloh et al., 2015; Armstead and Vavilala, 2019). It was found that cerebral glucose utilization in some brain regions, including the hippocampus, varied with the phases of the estrus cycle in uninjured, normal cycling female rats compared to male rats (Nehlig et al., 1985). It has also been shown that adult females have greater CBF than age-matched males; CBF diverges in the sexes around mid-puberty when estrogen levels rise in females, but CBF eventually drops to male levels around the age of 65 years old, around/after menopause when female hormone levels are low (Aanerud et al., 2017; Prins, 2017). This suggests glucose utilization in the brain is influenced by female sex steroids and would be an important topic to study after TB (Prins, 2017). Studies performed in young (~20 years old) male and female athletes with and without a history of concussion (HOC) showed men with HOC had significantly lower CBF than men without HOC. There was no significant difference between CBF of women with or without HOC; however, women who received multiple concussions had lower CBF than women who only received one (Hamer et al., 2020).

Following rodent TBI, there is an initial hypermetabolic state that involves increased extracellular potassium, glutamate, and calcium accumulation, which drives increased glucose utilization in the brain (Greco et al., 2020). The hypometabolic state occurs about 6 h post-injury in rats and the duration of this phase often varies with injury severity. The hypometabolic state is associated with decreased glucose utilization and mitochondrial dysfunction from altered ionic flux (Greco et al., 2020). Estrogen has been shown to play a role in promoting glucose utilization by facilitating glucose transport and increasing the expression or activity of key enzymes involved in glycolysis and the tricarboxylic acid (TCA) cycle (i.e., pyruvate dehydrogenase complex subunits, hexokinase, phosphofructokinase) in healthy neurons through activation of nuclear, plasma membrane-bound, and/or mitochondrial estrogen receptors (ERs; reviewed in Brinton, 2008). However, administration of exogenous glucose during the hypometabolic phase (6–9 h post-CCI) resulted in worsened mitochondrial respiratory control ratio (RCR) after severe CCI in both male and female rats, but administration of glucose during the hypermetabolic (0–3 h post-CCI) only worsened RCR in male rats after injury. The authors of this study hypothesized that females did not appear to be as metabolically impaired as males after the addition of glucose because it may not be considered a “stressor” in the females like it might be in the males after injury (Greco et al., 2020). Alternatively, administration of β-hydroxybutyrate (BHB) during the hypometabolic period increased mitochondrial respiration and Acetyl CoA content in injured males but had detrimental effects in females indicating the switch to ketone bodies as a fuel source may be harmful to female metabolic recovery after TBI. However, female cerebral metabolism has yet to be fully characterized after TBI.

## Discussion and Conclusion

There is limited overlap in available data regarding sex differences in traumatic brain injury and data on the effects of various sex hormones on mitochondrial function (see Table 1). To better understand the complex nature of female outcomes after TBI, it is vital to study the role of mitochondria. Mitochondria are important regulators of cellular homeostasis, calcium handling, and energy production so they are important therapeutic targets of interest to prevent further damage after injury. Most of what we know about mitochondrial bioenergetics, oxidative stress, and glucose utilization following TBI was studied in males, so to fill the gaps we need to conduct these experiments in female models, as well. This mini-review presents the available data utilizing the control cortical impact (CCI) model, summarized in Table 1 and Figure 1B, and hopefully reveals the gaps in knowledge we as scientists need to address in the field of mitochondria and TBI to be able to study and treat both males and females effectively. It is hypothesized that these mitochondrial changes represented in Figure 1B are influenced by female ovarian hormones (i.e., estrogen and progesterone) and testosterone, which is summarized by the studies in Table 1. One limitation in this field is the failure to apply other methods to model TBI for mitochondrial analyses in both sexes. Other injury mechanisms like a blast injury, weight drop injury, or rotational injury can be utilized to mimic the heterogeneity seen in the human TBI population; these injuries can then be applied to better understand mitochondrial dysfunction in both sexes (Saatman et al., 2008; Yonutas et al., 2016).

**Table 1:** Summary of literature in sex differences in mitochondrial dysfunction following TBI.

Summary of Sex Differences in Mitochondrial Bioenergetic Function after TBI				
Reference	Species (Age; Alteration)	TBI Model and Severity	Male Result	Female Result
Greco et al. (2020)	Rat (adult)	CCI; severe (2 mm depth)	Decreased State III respiration and RCR compared to sham.	Decreased State III and State V respiration, but no change in RCR compared to sham.
Robertson et al. (2006)	Rat (adult; OVX)	CCI; moderate-severe (1.5 mm depth)	-	Progesterone (25 ng/ml) improved RCR after CCI relative to sham.
Robertson and Saraswati (2015)	Rat (PND 17–21)	CCI; moderate-severe (1.5 mm depth)	Progesterone (10 mg/kg) improved RCR after CCI similar to sham.	CCI did not significantly reduce RCR compared to sham; progesterone treatment did not significantly improve RCR compared to the other groups.
Carteri et al. (2019)	Mouse (adult)	CCI; severe (2 mm depth)	Testosterone (15 mg/kg) improved State III respiration (CI) and maximum OxPHOS after CCI similar to sham; Testosterone improved ΔΨ_m_ after CCI similar to sham.	-
*CCI = controlled cortical impact; State III respiration = glutamate, malate, and ADP (Greco et al., 2020), pyruvate, malate, glutamate, and ADP (Carteri et al., 2019); RCR = (State III/State IV respiration) a general marker of mitochondrial health and coupling ability; OxPHOS = Carteri et al., 2019 defined it as maximal oxidative phosphorylation capacity after additions of pyruvate, malate, glutamate, and succinate in the presence of saturating ADP; ΔΨ_m_ = mitochondrial membrane potential*.				
**Summary of Sex differences in Mitochondrial Excitotoxicity, Oxidative Stress, and Lipid Peroxidation After TBI**				
**Reference**	**Species (Age; Alteration)**	**TBI Model and Severity**	**Male Result**	**Female Result**
Greco et al. (2020)	Rat (adult)	CCI; severe (2 mm depth)	Increased peroxide production after CCI similar to sham.	No change in peroxide production after CCI compared to sham.
Robertson and Saraswati (2015)	Rat (PND 17–21)	CCI; moderate-severe (1.5 mm depth)	Progesterone (10 mg/kg) prevented CCI-induced reduction in glutathione compared to uninjured control; uninjured male mitochondria had significantly lower levels of glutathione than uninjured female mitochondria.	Progesterone did not prevent CCI-induced glutathione reduction in brain mitochondria after injury compared to uninjured control; mitochondria from uninjured females had higher glutathione levels than that of uninjured males.
Carteri et al. (2019)	Mouse (adult)	CCI; severe (2 mm depth)	Testosterone (15 mg/kg) improved baseline peroxide production after CCI similar to sham; Testosterone attenuated peroxide production after respiratory substrate administrations, but was still increased compared to sham; Testosterone reduced total ROS levels similar to sham.	-
*CCI = controlled cortical impact; ROS = reactive oxygen species; GCS = Glasgow Coma Scale; CSF = cerebrospinal fluid*.				
**Summary of Sex Differences in Glucose Metabolism after TBI**				
**Reference**	**Species (Age; Alteration)**	**TBI Model and Severity**	**Male Result**	**Female Result**
Greco et al. (2020)	Rat (adult)	CCI; severe (2 mm depth)	Exogenous glucose administration during both the hyper- and hypometabolic states after CCI worsened mitochondrial RCR similar to vehicle CCI.	Exogenous glucose administration during hypometabolic state after CCI worsened mitochondrial RCR compared to sham and vehicle CCI; No significant impairment in RCR with glucose administration during hypermetabolic state similar to sham and vehicle CCI.

*CCI = controlled cortical impact; RCR = (State III/State IV respiration) a general marker of mitochondrial health and coupling ability; hypermetabolic state = 0–3 h post-CCI; hypometabolic state = 6–9 h post-CCI (Greco et al., 2020)*.

## Author Contributions

PS developed the concept for this review. OK researched and compiled the literature for this manuscript and wrote the review. PS and OK both revised the manuscript for submission. All authors contributed to the article and approved the submitted version.

## Conflict of Interest

The authors declare that the research was conducted in the absence of any commercial or financial relationships that could be construed as a potential conflict of interest.

## Publisher’s Note

All claims expressed in this article are solely those of the authors and do not necessarily represent those of their affiliated organizations, or those of the publisher, the editors and the reviewers. Any product that may be evaluated in this article, or claim that may be made by its manufacturer, is not guaranteed or endorsed by the publisher.
